# Associations between social isolation, pro-social behaviour and emotional development in preschool aged children: a population based survey of kindergarten staff

**DOI:** 10.1186/s40359-014-0044-1

**Published:** 2014-10-15

**Authors:** Louise Marryat, Lucy Thompson, Helen Minnis, Phil Wilson

**Affiliations:** Institute of Health and Well-being, University of Glasgow, Caledonia House, Royal Hospital for Sick Children (Yorkhill), Glasgow, G3 8SJ UK; Centre for Rural Health, University of Aberdeen, Centre for Health Science, Old Perth Road, Inverness, IV2 3JH UK

**Keywords:** Social isolation, Peer relations, Preschool, Kindergarten, Friends, Emotional development, Pro-social skills

## Abstract

**Background:**

The impact of peer relationships has been extensively reported during adolescence, when peer influence is generally considered to be at its greatest. Research on social isolation during childhood has found associations with school achievement, future relationships and adult mental health. Much of the evidence is derived from either parent or child-rated assessment of peer relationships, each of which have their limitations.

**Methods:**

We report findings from Goodman’s Strengths and Difficulties Questionnaire (SDQ), completed by staff in preschool establishments for over 10,000 children in their preschool year (aged 4–5), linked with routine demographic data. Correlations between scores and demographics were explored. Regression models examined the independent relationships between three social isolation variables, taken from the SDQ Peer Relationship Problems, Pro-social Behaviour and Emotional Symptoms subscales, controlling for demographics.

**Results:**

There were substantial overlaps between problem scores. Regression models found all social isolation variables to be significantly correlated with social and emotional functioning. Different types of social isolation appeared to relate to different psychological domains, with unpopularity having a stronger relationship with poor pro-social skills, whereas being solitary was more strongly linked to poorer emotional functioning.

**Conclusions:**

Social isolation does have a significant association with reported child social and emotional difficulties, independent of demographic characteristics. The analysis highlights the complexity of measuring social isolation in young children. Different types of social isolation were found to have relationships with specific areas of social and emotional functioning.

## Background

There is a long history of research on peer relationships in childhood in the field of developmental psychology (Bukowski & Adams 2005). Social isolation ‘is concerned with the objective characteristics of a situation and refers to the absence of relationships with other people’ (De Jong Gierveld et al. 2006). Social isolation in children has traditionally been defined by either rejection by peers or by solitary play, or a combination of the two. In his study of social isolation in children, Gottman described five types of children: children who were well accepted by their peers, children who were rejected by their peers, children who had highly negative interactions with their teacher, children who interacted frequently with peers and finally, children who were frequently tuned out or off-task when alone. This latter group of children had poor levels of acceptance among their peers whilst simultaneously having high levels of shyness, anxiety and fearfulness (Gottman 1977).

The preschool period is a key time for children to develop social skills that will allow them to be socially competent individuals and prepare them for school. Social isolation in childhood is important because of the long-lasting impacts it has been evidenced to have: social isolation has been found to be associated with poor performance at school, problematic later relationships, criminal behaviour and internalizing and externalizing problems both in later childhood and in adulthood (Hymel et al. 1990; Bukowski & Adams 2005; Gazelle 2006; Laursen et al. 2007; Spinrad et al. 2004; Buhs et al. 2006). However, there is also an argument that social isolation, particularly in the preschool period, is not necessarily problematic in itself, but rather may reflect a personal preference of some children to play alone (Hinde et al. 1993). Furthermore, for children with characteristics which put them at risk of being victimised, social isolation may act as a protective factor, with increasing numbers of friends for these children leading to poorer internalising outcomes (Bukowski & Adams 2005).

In addition, the reverse of social isolation, having friends, may positively moderate the impact of family adversity and the effect of harsh punishment on later externalising behaviours (Criss et al. 2002). In a Finnish study, friendship at age seven moderated the relationship between social isolation and internalising and externalising behaviours at age nine (Laursen et al. 2007).

However, much of the previous work with preschool aged children has either used observational data or peer nominations (otherwise known as sociometric status), each of which presents problems of interpretation. For example, the Hinde and colleagues paper observed children’s interactions and play at home and at preschool (Hinde et al. 1993). The difficulty with observational data is that they rely on an ‘outsider’ being present to monitor child interactions, which can unintentionally alter behaviour, an example of the Hawthorne Effect (Mays & Pope 1995). Sociometric status research was brought to the fore in the 1980s: children were asked to said how much they liked or disliked other children in the class, and then using class ratings, children are classified into popular, rejected, neglected, average or controversial children (Coie et al. 1982). Peer nominations may be confusing though for preschool children who are not always able to distinguish between who they are friends with and who they would like to be friends with (Hinde et al. 1993). The current study, by contrast, used teacher-rated Goodman’s Strengths and Difficulties Questionnaires (SDQ) (Goodman 1997), completed for all children in preschool establishments (kindergarten/nursery) in Glasgow City.

Evidence has also shown that other factors, such as the characteristics of the child and their family, are associated with pro-social behaviours and emotional symptoms in early childhood. In particular, experiencing poverty has been strongly associated with both current and later social and emotional functioning (Brooks-Gunn & Duncan 1997; Costello et al. 2003). Gender has been evidenced to affect social and emotional development, with boys having more difficulties in early childhood, but with depression and anxiety in girls becoming dominant in adolescence (Cohen et al. 1993; Ford et al. 2003). Other factors which have been evidenced to be associated with social and emotional functioning are ethnicity (Ford et al. 2003; Goodman et al. 2010; Bradshaw & Tipping 2010), being Looked After (under the supervision of the state) (Richardson & Lelliott 2003; Ford et al. 2007; Stanley et al. 2005; McAuley & Davis 2009; Minnis et al. 2006), parenting (Stewart-Brown & Schrader-Mcmillan 2011; Bayer et al. 2006) and the neighbourhood in which the child lives (Edwards & Bromfield 2009; Colder et al. 2006). The current study will attempt to taken into account these characteristic factors in the analysis as far as possible.

The current study hypothesised that children who were socially isolated at preschool age would also concurrently experience poorer social and emotional functioning. The research had 3 key aims:

1. To describe the prevalence of social, emotional and behavioural difficulties in preschool aged children using teacher-rated SDQs.
2. To explore overlaps between different areas of social, emotional and behavioural difficulties at preschool.
3. To investigate the associations between social isolation and pro-social behaviours, and between social isolation and emotional symptoms, at preschool.

## Methods

### Procedure

This paper uses the combined data from three years of preschool data collection (2010 to 2012) conducted as part of the Evaluation of the Glasgow City Parenting Support Framework (University of Glasgow 2013). As these data were collected as part of an evaluation, the study did not require ethical approval (Health Research Authority 2013).

In order to assess social, emotional and behavioural functioning at school entry, the SDQ (Goodman 1997) was administered as part of the routine process of transition for children about to start school in the city. In early 2010, 2011 and 2012, Child Development Officers (nominated staff members) within preschool establishments were asked to complete SDQs, alongside standard demographic information for every child eligible to start school in the subsequent August (White et al. 2013). Parents were informed that data were being collected and were able to opt out if desired.

The study involved 115 Local Authority nurseries and 87 ‘Partnership’ nurseries in Glasgow City. The latter are independent and voluntary sector nurseries from which the Council commissions places for children. Even though each child is entitled to attend an early years’ establishment from the age of three, attendance is not compulsory. Attendance in Glasgow varies from year to year: in 2011, 90.2% of eligible children attended a funded preschool place in the year prior to starting school, whereas in 2012, 82.5% attended. This is consistently lower than national average figures for subsidised attendance at a preschool establishment: 98.9% in 2011 (Scottish Government 2011) and 95.1% in 2012 (Scottish Government 2012a).

### Participants

Between 2010 and 2012, 10,873 forms were returned for preschool children, comprising 68% of the preschool population in Glasgow city. Fifty-two percent of children in the study were male and 48% were female. Children living in the most deprived neighbourhoods were well represented in the study: 62% of children with SDQs returned lived in the most deprived quintile, compared with 49% of the Glaswegian population overall (using the Scottish Index of Multiple Deprivation (SIMD - 2009 Scottish quintiles)) (Scottish Government 2012b). In the current sample, 2.3% children (n. = 251) were reported to be under local authority supervision, being looked after at home, away from home or, for a small minority, previously looked after. With respect to ethnicity, 72% of children were white and 28% were non-white, though it should be noted that there were substantial amounts of missing data for this field.

### Missing data

SDQ scores were missing for some 30% of children with funded preschool places in Glasgow, as well as for the 15% of all children in Glasgow City with no funded preschool place. Some data were also missing for variables within cases. Full data were available on 6343 children (58.3%) within the database. The quantity of individual missing data was greatest for ethnicity (n. = 3774) and postcode (n. = 1331). In addition between 30 and 32 cases were missing from each SDQ subscale, respectively.

In order to get a gauge of whether the missing children from the sample were demographically different, postcodes of the children in sample were compared with postcodes of all children of the appropriate age living in Glasgow City (from health service data). In comparison with all children in the city, children in the sample were more likely to live in an area of higher deprivation than others: 27% of children in the sample lived in the most deprived SIMD decile of deprivation, compared with 24% in the preschool aged population (Barry et al. 2014). Until the children reach school, at which point education is compulsory, it is difficult to assess other differences between the sample and all children in the year group.

### Measures

*Social and emotional problems* were measured using the SDQ (Goodman 1997), a brief behavioural screening questionnaire which produces sub-scale scores for Peer Relationship Problems, Emotional Symptoms, Hyperactivity/inattention, Conduct Problems (the four of which are summed to produce a total difficulties scale) and a positively rated Pro-social Behaviours scale (Table 1). There are two versions of the SDQ: a 4–16 year old version, and a 3–4 year old version, the latter of which contains two ‘softer’ items in the Conduct Problems scale. This study used the 4–16 year old version in 2010 and 2011, and then the 3–4 year old version (following concerns from nursery staff about the appropriateness of the 4–16 version for preschool aged children) in 2012 (White et al. 2013). All multivariate analyses are adjusted for year of completion in order to control for cohort effects.

**Table 1:** **Goodman’s strengths and difficulties questionnaire: examples of domains**

**Domain**	**Examples**
**Emotional Symptoms**	Many fears, easily scared; often complains of headaches/tummy aches; Many worries
**Conduct Problems**	Frequent temper tantrums; often fights with other children; Can be spiteful
**Hyperactivity/Inattention**	Restless, overactive; easily distracted; Constantly fidgeting or squirming
**Peer Relationship Problems**	Rather solitary; picked on or bullied; gets on better with adults than children
**Pro-social Skills**	Considerate of other people’s feelings; shares readily; helpful is someone is hurt

Social isolation was captured in three individual items of the SDQ, which comprise part of the Peer Relationships Scale, namely, being deemed to be ‘rather solitary’ (relating to the aspect of social isolation around solitary play), not having ‘at least one good friend’ and not being ‘generally liked by peers’ (the latter two items relating to rejection by peers).

*Area Deprivation* was measured using the Scottish Index of Multiple Deprivation Quintiles (Sameroff 1998), which is a composite measure of neighbourhood disadvantage comprising 38 indicators of deprivation across seven domains: income, employment, health, education, skills and training, housing, geographic access and crime. The data were analysed by SIMD quintile, with quintile 1 being the most deprived and quintile 5 the least.

### Analysis plan

SDQ scores were described in terms of range, mean and standard deviation, and compared with UK norms. A correlation matrix of study variables was produced in order to examine the bivariate correlations between risk factors and SDQ scores. Correlations between Pro-social Behaviour/Emotional Symptoms scores and other scales were further examined and the Peer Relationship Problems scale was broken down into its constituent parts so as to further explore individual items pertaining to social isolation.

An ecological approach (Bronfenbrenner 1986) was taken to the analysis: investigating social isolation in the context of child, family and wider environmental factors. Using MLwiN, single level and multi-level empty models were fitted in order to explore whether multilevel analysis was appropriate in this case. Models were fitted for two outcomes: having abnormal Pro-social skills and having abnormal Emotional Symptoms, respectively. The models explored differences at two levels: children within nurseries. Neither model showed statistically significant nursery level differences in the empty two-level models. Furthermore, no confidence intervals on the residuals for nurseries were significantly different from the norm, further indicating no statistically significant differences between nurseries. Therefore, analysis reverted back to single level models using SPSS. Two logistic regression models were fitted on each scale respectively. The first of these controlled solely for the effects of socio-demographic and environmental characteristics, such as child sex, ethnicity and neighbourhood deprivation. The second model incorporated individual items from the Peer Relationship Problems scale whilst controlling for demographic and environmental risk factors.

## Results

Table 2 describes the range, mean and standard deviation for Glasgow preschoolers’ scores on each scale, and compares these to the UK norms for teacher-completed 5–10 year olds^a^ (Green et al. 2006). Glasgow SDQ scores map on well to the UK scores, though it should be noted that scores vary considerably between the ages of 5 and 10, and the Glasgow children are a little younger at 4–5. Thus scores only give a rough indication of underlying differences between the two datasets.

**Table 2:** **Description of glasgow SDQ Scores and a comparison with UK SDQ data from the British mental health survey of children and young people, 2004**

**Scale**	**Range**	**Mean – Glasgow (SD)**	**Mean – UK 5-10 yr olds (SD)**
**Emotional Problems**	0-10	1.1 (1.7)	1.5 (1.9)
**Conduct**	0-10	0.8 (1.4)	0.9 (1.6)
**Hyperactivity**	0-10	2.7 (2.6)	3.0 (2.8)
**Peer Relations**	0-10	1.5 (1.8)	1.4 (1.8)
**Total Difficulties**	0-36	6.0 (5.5)	6.7 (5.9)
**Pro-social**	0-10	7.4 (2.6)	7.3 (2.4)
***Base***		*10840*	*4801*

SDQ scores can be banded into groups using the published cut-offs (Goodman 2013). These bands are ‘Normal’, ‘Borderline’ and ‘Abnormal’. The cut-offs are set so that Abnormal and Borderline groups should produce 10% in each group, respectively, in a normal population. Work by Goodman et al. on the British Mental Health Study data has shown that the predictive value of teacher ratings, alone, in the abnormal category vary from 15.9% predicting any anxiety disorder, to 49.3% predicting any hyperkinetic disorder and 47.9% predicting any conduct-oppositional disorder (Goodman et al. 2000a).

**Figure 1:**
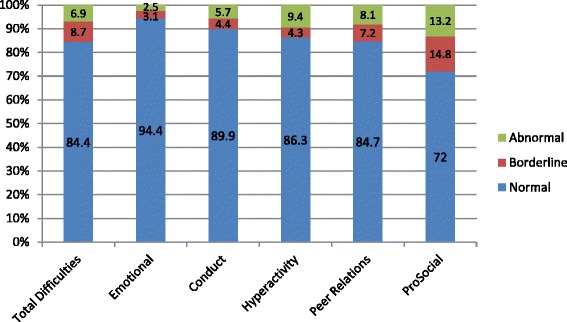
**SDQ banded scores for Glasgow pre-schoolers 2010-2012.**

The vast majority of children at this age in Glasgow City were classified as ‘normal’, from 72% on the Pro-social Behaviour scale, to 94.4% on the Emotional Symptoms scale. Furthermore, almost 9% are described as ‘borderline’ on the Total Difficulties scale, with an additional 6.9% falling into the ‘abnormal’ group. The highest proportion of preschool children in the ‘abnormal range’ on an individual scale is on the Pro-social Behaviour scale, where 13.2% children were classified as having ‘abnormal’ development, followed by the Hyperactivity/Inattention scale, where the proportion was 9.4% (Figure 1).

A correlation matrix of study variables is presented in Table 3. All SDQ subscale scores were significantly correlated. The Pro-social Behaviour score was strongly correlated with the Hyperactivity/Inattention and Conduct Problems scores. There was also a reasonably strong positive correlation between the Hyperactivity/Inattention and Conduct Problems scales (p = .58) and a fairly strong negative correlation between Pro-social Behaviours and Peer Relationship Problems scores (p = −.49).

**Table 3:** **Correlations between study variables**

	**1**	**2**	**3**	**4**	**5**	**6**	**7**	**8**	**9**
**1. Emotional**	1.00								
**2. Conduct**	.19**	1.00							
**3. Hyperactivity**	.24**	.59**	1.00						
**4. Peer relations**	.40**	.33**	.38**	1.00					
**5. Pro-social**	-.23**	-.60**	-.67**	-.49**	1.00				
**6. Deprivation (SIMD)**	-.09**	-.07**	-.09**	-.05**	.06**	1.00			
**7. Non-white a.**	-.06**	-.04**	.01^	.09**	-.07**	.09**	1.00		
**8. Looked After a.**	.07**	.08**	.09**	.04**	-.04**	-.07**	-.07**	1.00	
**9. Female a.**	.03*	.17**	.25**	.06**	-.24**	.03**	.01^	-.01^	1.00

^p < .10; *p < .05;**p < .01.

a. Spearman Correlations.

Correlations between scores and demographic variables were weak. Looked After status (being under the supervision of the state) was positively correlated with all SDQ scores, except the (positively scored) Pro-social Behaviour score. Affluence of area of residence was correlated with all scores, except the Pro-social Behaviour scale. Non-white children scored better on Emotional Symptoms and Conduct Problems scores, but worse on Peer Relationship Problems and Pro-social Behaviour, and results were non-significant on the Hyperactivity/Inattention scale. Correlations between gender and ethnicity/Looked After status, respectively, were only significant to the *p* < 0.10 level.

Overlaps between abnormal category scores on two scales were also fairly strong: on the Pro-social Behaviour scale, two fifths of children who were in the abnormal group were also in the abnormal Hyperactivity/Inattention group, whilst 31.1% were also in the Peer Relationship Problems abnormal group. In contrast, just 7.4% of children who were in the abnormal group on the Pro-social Behaviour scale were in the abnormal group on the Emotional Symptoms scale, though it should be noted that there were few children of this age in the abnormal Emotional Symptoms group overall.

**Figure 2:**
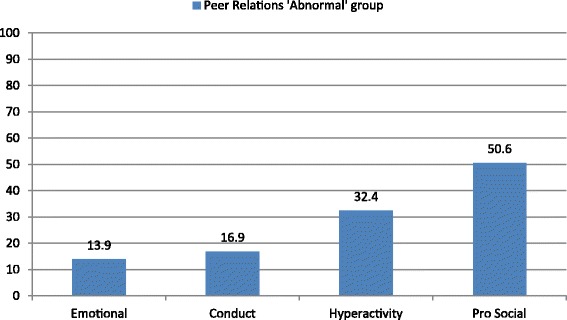
**Children scored as ‘abnormal’ on the Peer Relationship Problems scale, by ‘abnormal’ scores on the remaining SDQ scales.**

Among children who scored in the abnormal range on the Peer Relationship Problems scale, half also scored in the abnormal range of the Pro-social Behaviour scale (Figure 2), suggesting that a considerable proportion of children who experience problems with peers, also show few pro-social behaviours. In addition, a third of children who were in the abnormal group on the Peer Relationship Problems scale, also fell into the abnormal group on the Hyperactivity/Inattention scale. There was far less overlap between children in the abnormal Peer Relationship Problems group and the Conduct Problems and Emotional Symptoms groups, respectively.

**Figure 3:**
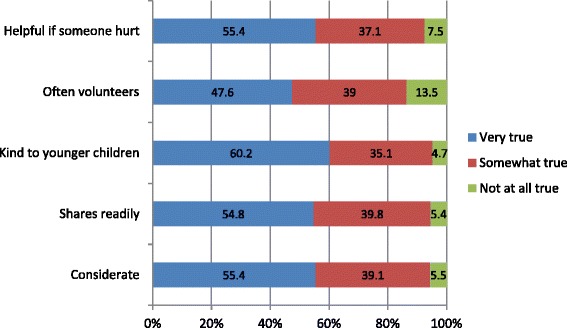
**Breakdown of responses to ‘Pro-social scale’.**

The largest proportion of difficulties was in the Pro-social Behaviours Scale, which is a positively scored scale, and hence abnormal development here indicates an absence of certain social qualities, rather than an exhibition of problem behaviours. When the scale is broken down into its constituent parts, it is apparent that fewer children are said to ‘often volunteer to help others’ (13.5% saying that this is not at all true) and to be ‘helpful if someone is hurt, upset or feeling ill’ (7.5% being ‘not at all’ like this). This may be a reflection of a lack of maturity in some children of this age, rather than being illustrative of a fundamental social problem. It is also clear when looking at the individual scale items that a substantial proportion of children fall into the ‘somewhat like this’ group on all categories, which will add to a poorer score overall (Figure 3).

### Multivariate analysis

Looked After status, SIMD quintile, ethnicity, gender and cohort were entered into a forward stepwise regression model in order to ascertain if any had an independent correlation with abnormal Pro-social Behaviours (Table 4) or Emotional Symptoms (Table 5), as opposed to being ‘borderline’ or ‘normal’. Being male, not white and having ever been under the supervision of the local authority were related to being in the abnormal Pro-social Behaviours group, whilst living in a more affluent neighbourhood was related to not being in the abnormal Pro-social Behaviours group. There were no significant differences between Total Difficulties, Pro-social Behaviours or Emotional Symptoms scores, respectively, obtained for the 2010, 2011 and 2012 cohorts. The demographic Pro-social Behaviours model explained only c.7% of the variation.

**Table 4:** **Coefficients from binary logistic regression models predicting ‘abnormal’ pro-social outcomes at preschool**

**Predictors**	**Model 1**	**Model 2**
**Child Sex**	1.12 (.08)	1.0 (.09)
**Deprivation Quintile**	-.16 (.04)	NS
**Looked After Status**	.90 (.22)	.59 (.26)
**Ethnicity**	.46 (.08)	NS
**Cohort**	NS	-.13 (.56)
**Not Generally liked**	-	1.7 (.09)
**Not have a friend**	-	.63 (.07)
**Rather solitary**	-	.34 (.07)
**Externalising Symptoms**	-	-
***Rsq***	*0.07*	*0.36*
***Bases***	*6349*	*6343*

**Table 5:** **Coefficients from binary logistic regression models predicting ‘abnormal’ emotional outcomes at preschool**

**Predictors**	**Model 1**	**Model 2**
**Child Sex**	.26 (.11)	NS
**Deprivation Quintile**	-.19 (.06)	-.14 (.06)
**Looked After Status**	1.0 (.26)	.70 (.29)
**Ethnicity**	NS	-.56 (.14)
**Cohort**	NS	NS
**Not Generally liked**	-	.72 (.12)
**Not have a friend**	-	.25 (.10)
**Rather solitary**	-	.93 (.09)
**Externalising Symptoms**	-	-
***Rsq***	*0.02*	*0.17*
***Bases***	*6349*	*6343*

The demographic model predicting whether a child is in the abnormal Emotional Symptoms group contained three independently correlated variables: gender, area deprivation and looked after status. Neither ethnicity nor cohort was significant in the model. This model only explained c.2% of the variation.

Model two presented results from multivariate regression for abnormal Emotional Symptoms and Pro-social Behaviours scores, adding social isolation variables to the socio-demographic variables. All three social isolation variables (being rather solitary, not having at least one good friend and not being generally liked by peers) were significant contributors to both the Pro-social Behaviours and Emotional Symptoms models. The Pro-social Behaviours model explained c.36% of variation in the model, suggesting that the three social isolation variables alone account for around 30% of the variation. Once social isolation effects were controlled for, deprivation and ethnicity were no longer significantly correlated to Pro-social Behaviour outcomes, and the effect of being male and ever Looked After declined. In contrast, a difference between the years could now be seen, with later years being correlated with being slightly less likely to be in the abnormal group.

Social isolation variables had a weaker relationship with Emotional Symptoms: once demographics and social isolation variables were controlled for, the model explained 17% of the variation between cases, compared with 2% when only demographics were modelled. Once social isolation was accounted for, gender was no longer significantly associated with Emotional Symptoms. However, ethnicity became significant once social isolation was controlled for, so that being non-white was related to a decreased likelihood of being in the abnormal Emotional Symptoms group.

Although all three individual markers of social isolation were significant in both models, the strength of the relationship between individual markers differed markedly depending on the outcome being investigated, over and above demographics. Not being ‘generally liked’ by other children had the strongest correlation with Pro-social Behaviour scores, with being classified as not having ‘at least one friend’ also being related to an increased risk of being in the abnormal Pro-social Behaviours group. In contrast, in relation to abnormal Emotional Symptoms scores, the strongest correlation was with being ‘rather solitary’, followed by not being ‘generally liked’.

## Discussion

The preschool environment aims to encourage social interaction with peers and the development of key social and emotional skills before proceeding to primary school (Bridges et al. 2004). Indeed, the policy of provision of free preschool education in Scotland is considered a ‘key element’ in increasing social ‘solidarity and cohesion’ (Scottish Government 2009) through developing these early skills. Some children are nevertheless still passively or actively socially isolated even within this environment. Previous research in the field has reported correlations between social isolation in early to middle childhood and a range of poor outcomes, including internalising and externalising behaviours e.g. (Bukowski & Adams 2005). Research conducted with younger children however raises methodological issues concerning the measurement of both social isolation and peer relationships (Hinde et al. 1993). We have addressed the question of the role of social isolation in preschool children’s social and emotional skills in a large preschool sample, as rated by kindergarten staff who knew the children well, taking account of the wider demographic context. It was hypothesised that being socially isolated may hinder pro-social and emotional development in pre-school aged children.

Support was found for the hypothesis that being socially isolated is associated with poorer social and emotional functioning in preschool. Direct associations were found between indicators of social isolation, and pro-social skills and emotional symptoms, respectively, even once demographic characteristics of the child were controlled for. Whilst these relationships clearly existed, the nature of the associations varied. Unpopularity in the preschool peer group had a strong independent association with having poor social skills, with the second strongest association being with children who did not have at least one good friend. It is intuitive that children with poorer pro-social skills may find it harder to interact successfully with their peers and that this lack of interaction prevents further development in such pro-social skills, as previously evidenced (Spinrad et al. 2004). This group of children may also include those with autistic spectrum disorders, who have been described as having ‘impaired social instinct’ (Wing 2006). However, other studies have shown that better developed social skills in kindergarten may be associated with poorer peer relations, due to children being better able to manipulate other children and use these skills in a negative way (Hoglund & Leadbeater 2004).

Demographic data were found to explain very little of the variance within the models. It is unfortunate that the database to which the study had access had few measures of family background, which meant that analysis was limited to data routinely collected by schools, which is primarily based around child rather than family characteristics. Deprivation was measured at a neighbourhood level, which may provide less explanatory power than household income or deprivation. Previous research has found that neighbourhood based variables rarely explain more than 10% of variance within models of child socio-emotional outcomes (Sellstrom & Bremberg 2006). Being male was a greater independent risk for abnormal Pro-social Behaviours than it was for Emotional Symptoms at this age, though it was significant for both. Australian findings using teacher rated SDQs with 4–5 year olds found that being male outweighed any effects of Socio-Economic Status on all subscales with the exception of the Emotional Symptoms scale (Davis et al. 2010).

Emotional Symptoms appeared to be related to different aspects of social isolation, in particular to being solitary and preferring to play alone, though being unpopular also had a relatively strong relationship with emotional problems in preschool. The difference in the association between these separate social isolation characteristics and various difficulties leads to the question of whether they are picking up on the same issue. Coplan highlighted the complexity of social isolation, reporting on three different types of social isolation: shyness, social disinterest and social avoidance. Although there is little research around socially avoidant children (those who both desire solitude and actively seek to avoid social interaction), the little which has been done suggests that these children may be at particular risk for depressive symptoms and poorer overall well-being, which may be reflective of the emotional model findings above. Shyness on the other hand is said to be related to poorer social competence, lower self-esteem, anxiety and peer rejection, which may be more reflective of the children who score in the abnormal range on the pro-social scale and also on the unpopularity scale. The third group of children who were deemed to be ‘unsociable’ were found to interact less but otherwise had outcomes very similar to socially normal children (Coplan & Armer 2007).

Overall, this research highlights the importance of the development of peer relationships and the skills to successfully negotiate these early childhood relationships, which appears to be associated with other areas of social and emotional functioning. The results support the work of initiatives such as ‘nurture corners’, which take children in need of extra support out of the main nursery in small groups where they are encouraged to develop relationships and to constructively interact with their peers (Gerrard 2006), and the PATHS curriculum (Provide Alternative Thinking Strategies), which educates teaching staff to deliver a curriculum to enhance the social understanding and competence of children. In a pilot with preschool aged children, teachers rated children who had experienced the PATHS curriculum as less likely to be socially withdrawn by the end of the year, compared with their peers who had not had PATHS (Domitrovich et al. 2007). It may be that this side of skill development should be focused on more in the preschool stage in order to better equip children emotionally for school and later life.

The collection of such data on children’s social, emotional and behavioural difficulties at the preschool stage raises the question of whether this could and should be used to screen for psychiatric disorders. There are both reasons for and against this. The current rates of children receiving help for mental health problems have been found to be incredibly low, with a recent study showing that just 10% of children with such difficulties at age 4 receive any sort of help for this (Wichstrom et al. 2012). Goodman suggests that population SDQ-based screening for mental health problems could potentially double or treble the proportions of children receiving help (Goodman et al. 2000b). The universal aspect of the screen may also reduce the stigma of accessing such help (Mabelis & Marryat 2011). However, the positive predictive value of the SDQ is limited and screening may risk falsely identifying children (Goodman et al. 2000b). In addition, evidence has shown that labelling children with a disorder in a class setting may actually lead to poorer outcomes (Sayal et al. 2010). However, it would seem unethical to collect these data about children’s difficulties and not to do anything about them. Longitudinal data collection with these children across primary school will inform this argument by providing data on the continuity of children’s problems between preschool and the end of primary school.

### Strengths

This study combines three years of data from children in Glasgow City nurseries, resulting in large numbers of high quality data being available, which allows analysis into detailed areas, such as different types of social isolation, to be conducted. The SDQs were completed by nursery staff, in contrast to many previous studies which used child-based peer nominations or observations. This is an advantage as staff have been able to observe children over several months – data collection took place in the spring term – approximately 6–8 months after most children started preschool and thus the children and staff had time to get to know each other and settle in, and their ratings of the children are therefore not subject to the Hawthorne Effect in the same way. Being part of routine data collection meant that response rates were good and, as far as we are able to tell, there does not appear to be a response bias again those with problems or from disadvantaged backgrounds, as other parent-completion studies have found (Wolke et al. 2009).

### Limitations

Although the research sought to minimise some of the methodological limitations of previous studies of social isolation with children this age, this did not come without its own limitations. Nursery staff may have had different thresholds for ‘abnormal’ and ‘normal’ behaviour, and thus some nurseries may have produced ‘better’ results than others. The results were also collected by Education Services at Glasgow City Council which, while this made for good response rates, may have led some nursery staff to create more favourable impressions (or otherwise) of the children in their nursery, than if data were collected purely for research purposes. In addition, previous research has found that nursery staff may find it difficult to identify children with social and emotional difficulties (Giannakopoulos et al. 2014), though the use of the standardised SDQ should help this. Furthermore, the use of a single informant on the SDQ, rather than using multiple informants reduces the precision of the measure (Goodman et al. 2000b; Goodman et al. 2004). If resources had been available, the study would be enhanced by the collection of parent-rated SDQs in addition to the teacher-rated data.

One of the greatest limitations was the lack of family level variables, such as family type and household income, which were available, due to the data being part of routine data collection in Glasgow City Council Education Services. Evidence from previous studies show that family level variables may explain some of the variation in outcomes (Caughy et al. 2007; Ford et al. 2003; Bradshaw & Tipping 2010).

Furthermore, SDQ scores were missing for some 30% of children who attended preschool in Glasgow, as well as for the 15% of all children in Glasgow City who do not attend a funded preschool place. In comparison with overall postcode data received from National Health Service Greater Glasgow and Clyde, children in the nursery cohort are more likely to live in an area of higher deprivation, than children in the population eligible for school in each comparative year group (Barry et al. 2014). It is unclear whether the SDQ scores and correlations would be different for this missing group of children, although it is notable that deprivation quintile was not independently associated with social and emotional development once the social isolation variables were incorporated into the model, suggesting that this may not be the case.

## Conclusions

Social isolation appears to operate in different ways in relation to social and emotional development, with unpopular children having poorer social skills, whilst more withdrawn children had poorer emotional development. The research highlights the need for further investigation of different types of social isolation in young children, as they may lead to different problematic outcomes. Further research using longitudinal data in order to examine the direction of causality would be beneficial. Future research should attempt to use multi-informants so as to increase the accuracy of the measurement.

### Endnote

^a^SDQ norms come from a large national survey of child and adolescent mental health carried out by National Statistics in funded by the Department of Health. This representative British sample included 10,438 individuals aged between 5 and 15. Complete SDQ information was obtained from 10,298 parents (99% of sample), 8,208 teachers (79% of sample) and 4,228 11–15 year olds (93% of this age band).
